# A descriptive study on spatial and temporal distributions of genetic clusters of porcine reproductive and respiratory syndrome virus infecting pig sites in Quebec, Canada, between 2010 and 2019

**DOI:** 10.1186/s40813-024-00357-x

**Published:** 2024-01-25

**Authors:** Marie-Ève Lambert, Julie Arsenault, Jean-Charles Côté, Sylvie D’Allaire

**Affiliations:** 1 Laboratoire d’épidémiologie et de médecine porcine, Faculty of Veterinary Medicine, Université de Montréal, St. Hyacinthe, QC Canada; 2 Centre de recherche en infectiologie porcine et avicole - Fonds de recherche du Québec – Nature et technologies, Faculty of Veterinary Medicine, Université de Montréal, St. Hyacinthe, QC Canada; 3 Groupe de recherche sur les maladies infectieuses en production animale, Faculty of Veterinary Medicine, Université de Montréal, St. Hyacinthe, QC Canada

**Keywords:** Porcine reproductive and respiratory syndrome virus (PRRSV), Open reading frame 5 (ORF5), Molecular epidemiology, Genetic clusters, Quebec

## Abstract

**Background:**

The wide diversity of porcine reproductive and respiratory syndrome virus (PRRSV) strains combined with incomplete heterologous cross-protection complicates the management of the disease at both the herd and the regional levels. The objectives of this study were to describe the spatial and temporal distribution of various PRRSV genetic clusters infecting pig sites in Quebec, Canada, and to compare PRRSV regional diversity of wild-type sequences over the years.

**Materials and methods:**

A retrospective surveillance-based study was conducted on all pig sites which had PRRSV ORF5 sequences from field submissions transferred into the Laboratoire d'épidémiologie et de médecine porcine database from January 1, 2010 to December 31, 2019. A maximum likelihood phylogenetic tree inferred from multiple sequence alignment was used to identify genetic clusters. For each wild-type cluster gathering ≥ 15 sequences, the number of pig sites in which the cluster was detected per administrative region and per year were displayed on bubble charts and the spatiotemporal distribution of pig sites was illustrated using pie chart maps. A molecular analysis of variance was performed to compare PRRSV wild-type sequence diversity according to the administrative region for each year.

**Results:**

A total of 32 wild-type clusters gathering 1653 PRRSV2 sequences from 693 pig sites were described. Each cluster was detected on up to 132 pig sites and 7 administrative regions over the 10-year period. Annually, the mean (min–max) number of wild-type clusters detected in at least one pig site reached 24 (17–29). Some clusters remained localized on a few sites over time whereas others were widespread over the territory during a few or many years. For each year, regional differences were also observed in PRRSV diversity of wild-type sequences.

**Conclusions:**

The differences observed in both the spatiotemporal distributions of PRRSV clusters and in the regional diversity of wild-type sequences highlight the importance of ongoing provincial surveillance to improve collective PRRS management strategies.

**Supplementary Information:**

The online version contains supplementary material available at 10.1186/s40813-024-00357-x.

## Background

Porcine reproductive and respiratory syndrome (PRRS) is one of the most economically important endemic diseases in swine production, worldwide [4]. It is characterized by reproductive disorders in sows and respiratory diseases in pigs of all ages [27]. In Canada, the annual losses due to mortality and reduced growth caused by PRRS were estimated at $150 million [24].

The etiological agent of PRRS is a single-stranded, positive-sense RNA virus (PRRSV) of the *Arteriviridae* family, genus *Betaarterivirus* [15]. PRRSV strains are divided into two species, PRRSV1 (*Betaarterivirus suid 1*, the European type) and PRRSV2 (*Betaarterivirus suid 2*, the North American type) [3]. The PRRSV genome is 15.5 kb in length and encodes 10 open reading frames (ORFs) [11, 15]. ORF5 codes for the envelope glycoprotein GP5. It is 603 nucleotides in length and is the most variable genome region. It is widely used for PRRSV strain classification for both research and surveillance purposes [16, 19, 33]. A wide diversity of PRRSV2 ORF5 sequences was revealed among thousands of sequences gathered in Quebec, Canada [10, 19].

Since the swine immune cross-protection against heterologous PRRSV strains is incomplete [17], preventing the introduction of new strains in a herd is essential. Numerous transmission modes are involved in PRRSV transmission between herds, including direct contacts with infected pigs or semen, indirect contacts with contaminated fomites entering the barn [27, 28] and aerosols [9]. Consequently, prevention of PRRSV introduction at the herd level is challenging. Annually, up to 19% of Quebec swine breeding herds experience the introduction of at least one new PRRSV strain [18]. To proactively manage the disease, PRRS regional control and elimination (ARC&E) initiatives were established in Quebec [21], similar to what has been done elsewhere [1, 23].

At the regional level, a better understanding of the geographic distribution of pig sites infected by various PRRSV genetic clusters over time could be used to collectively improve PRRS management strategies, as they represent potential sources of infection for other herds. Hence, a region with pig sites infected by a limited number of clusters and free from other clusters could be protected by limiting pig movements or other contacts from farms located outside the aforementioned region. Likewise, the distribution of PRRSV genetic clusters could guide the partition of the territory into different ARC&E initiatives to facilitate the collective control. Insights into the regional genetic diversity of PRRSV strains circulating in pig sites could provide complementary information in that regard. The objectives of our study were to describe the spatial and temporal distribution of infected pig sites by various PRRSV wild-type genetic clusters in Quebec between 2010 and 2019 and to compare PRRSV regional diversity of wild-type sequences for each year.

## Methods

### Study design

A retrospective surveillance-based study was conducted on pig sites of the province of Quebec, Canada, covering the period from 2010 to 2019, inclusively. In 2019, there were 2445 commercial pig sites members of the Éleveurs de porcs du Québec (Québec Swine Producers Association) and registered in the Veille Sanitaire Provinciale database [6] and 7.1 million hogs were produced annually [5].

### Sequence and data collection from pig sites

All pig sites having PRRSV2 ORF5 sequences transferred into the LEMP-DB (Laboratoire d'épidémiologie et de médecine porcine; Swine Epidemiology and Medicine Laboratory-Database; Faculty of Veterinary Medicine, Université de Montréal, QC, Canada) between January 1, 2010, and December 31, 2019, were included in the current study. These sequences were from field samples submitted by the herd veterinarian within its regular herd follow-up and on a producer voluntary basis. Samples were sent to the Molecular Diagnostic Laboratory (Faculty of Veterinary Medicine) or to two other private laboratories in Quebec. PRRSV RNA extraction, reverse transcription-polymerase chain reaction (RT-PCR) and ORF5 sequencing were performed according to each laboratory’s routine protocols. Since 2010, through an agreement signed up by all Quebec swine veterinarians, sequences from the three diagnostic laboratories are weekly transferred to the LEMP-DB along with the date of sampling, the name of the farm and the name of the veterinarian. This information was used to extract the geographical coordinates of the production site from the province-wide swine health monitoring network database, Veille Sanitaire Provinciale (VSP) database [6].

### Classification of sequences into genetic clusters

PRRSV ORF5 sequences were classified into genetic clusters using a methodology previously described [19]. For consistency with genetic clusters previously identified in Quebec [19], the classification was run on all available sequences in the dataset from 1998 to 2019. For the purpose of classification, the PRRSV ORF5 sequences of four commercial PRRS vaccines were obtained from GenBank (https://www.ncbi.nlm.nih.gov/genbank/) and added to the LEMP-DB: Ingelvac PRRS® MLV (Boehringer Ingelheim Vetmedica, Inc., St. Joseph, MO), Ingelvac PRRS® ATP (Boehringer Ingelheim Vetmedica Inc.), Fostera® PRRS (Zoetis, Florham Park, NJ) and Prime Pac™ PRRS + (Merck Animal Health, Summit, NJ). Briefly, a multiple alignment was performed using Clustal Omega with default settings [29], and a maximum likelihood (ML) phylogenetic tree was inferred using Randomized Axelerated Maximum Likelihood (RAxML, Pthreads AVX version 8.2.8) with a GTRGAMMA evolutionary model [30]. A Python script was used to detect all genetic clusters with ≥ 70% rapid bootstrap (1000 iterations) branch support value and ≥ 15 sequences, while preserving their hierarchical structure [19]. Clusters corresponding to the genetic clusters previously identified [19] were assigned as such. When a previously identified cluster was now divided in sub-clusters, it was named after the previous cluster followed by a sequential suffix. A cluster was considered vaccine-like when it contained at least one of the four vaccine reference sequences previously mentioned. Other clusters or sequences were considered wild-types. From the 1998–2019 clustering results, sequences from the 2010–2019 study period were extracted and kept for the study.

### Spatial, temporal and spatiotemporal distribution of wild-type clusters

SAS software (version 9.4; SAS Institute Inc, Cary, NC) was used for data management and description. Only clusters with ≥ 15 sequences collected during the study period were kept for the analyses on clusters distribution. For each wild-type cluster, the number of pig sites in which the cluster was detected per year and per administrative region was displayed on bubble charts. The size of each bubble was proportional to the number of pig sites. The spatiotemporal distribution of cluster-infected pig sites was mapped in ArcGIS software (version 10; Esri, Redlands, CA). The regional county municipalities (MRC) and year were used as geographical and temporal units, respectively. For each genetic cluster, a pie chart was drawn to illustrate the years in which the cluster was identified in at least one pig site within the MRC.

### Comparison of genetic diversity of wild-type sequences between regions

Only pig sites located in administrative regions that had wild-type sequences from at least 5 sites in each year of the study period were included in the genetic diversity analyses. For each pig site, a single wild-type sequence per year was randomly selected in SAS. Following a multiple sequence alignment performed in Clustal Omega [29] as implemented in the msa package [2], a distance matrix was computed in the ape package [26] of R (version 4.2.2.). The distribution of the genetic distances between pig site sequences for each administrative region and year was illustrated using violin charts in ggplot2 package [31]. For each year, a molecular analysis of variance (AMOVA) [12] was used to assess the differences in genetic diversity of wild-type sequences between administrative regions, performed in the pegas R package [25]. In presence of a significant effect for a specific year (*P* < 0.005 to account for multiple testing), post-hoc pairwise comparisons between administrative regions were done using Bonferroni adjustment over all possible tests (*P* < 0.002).

## Results

### Distribution of submitted sequences

**Fig. 1:**
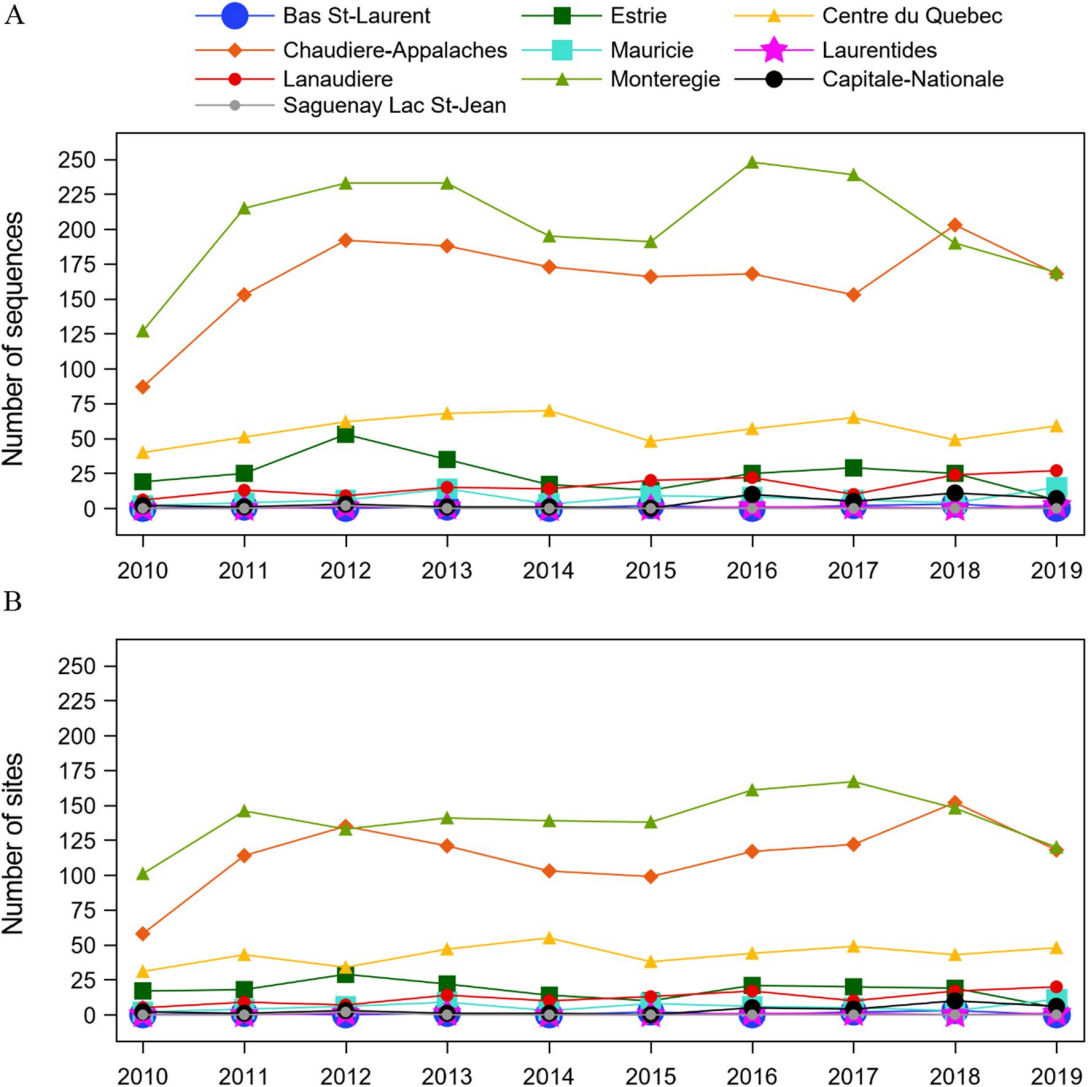
Yearly distribution of PRRSV sequences from pig sites with at least one sequence, by region. A total of 4796 PRRSV ORF5 sequences from 1334 pig sites located in 10 administrative regions in Quebec, Canada, were gathered during the study period (2010–2019). **A** Yearly number of PRRSV sequences submitted by administrative region. **B** Yearly number of pig sites from which at least one PRRSV sequence was submitted, by administrative region

A total of 4796 PRRSV2 sequences from 1334 pig sites located in 10 administrative regions and 49 MRC were collected between January 1, 2010, and December 31, 2019. The Monteregie and Chaudieres-Appalaches administrative regions had the largest number of sequences in all years (Fig. 1). The average (min–max) number of sequences per pig site over the study period was 4 (1–27). Between 213 and 439 sites had at least one sequence submitted in a given year, for an average of 329 sites per year. The average (min–max) number of sequences submitted per year was 466 (280–578).

### Classification of sequences in genetic clusters

**Fig. 2:**
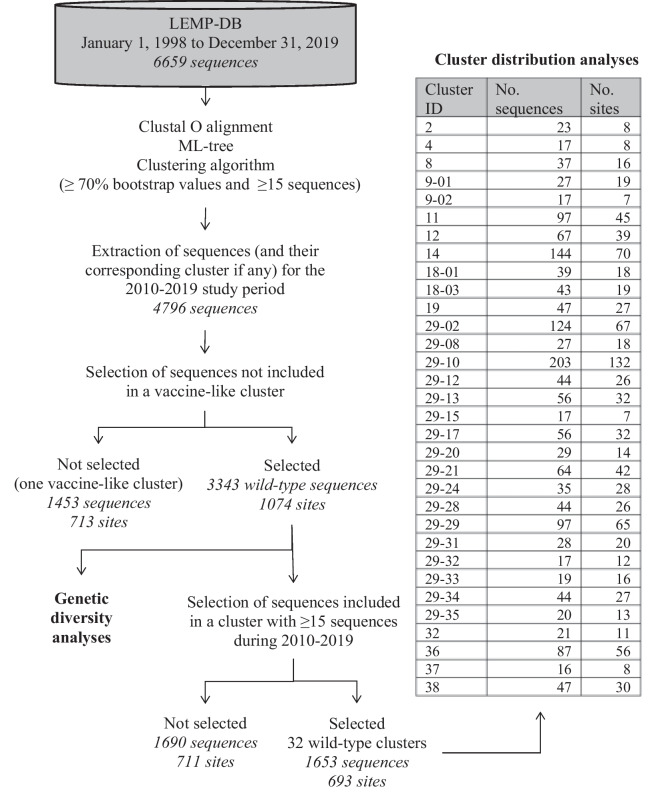
Flowchart of the clustering results for PRRSV sequences obtained from pig sites

PRRSV sequences were classified using the 6659 sequences transferred to the LEMP-DB from January 1, 1998, to December 31, 2019, with the addition of the four reference vaccine strains (Fig. 2). From the 4796 sequences collected during the study period, 32 wild-type clusters met the inclusion criteria (≥ 15 sequences during the study period) and were further described (Fig. 2).

### Description of wild-type genetic clusters

**Fig. 3:**
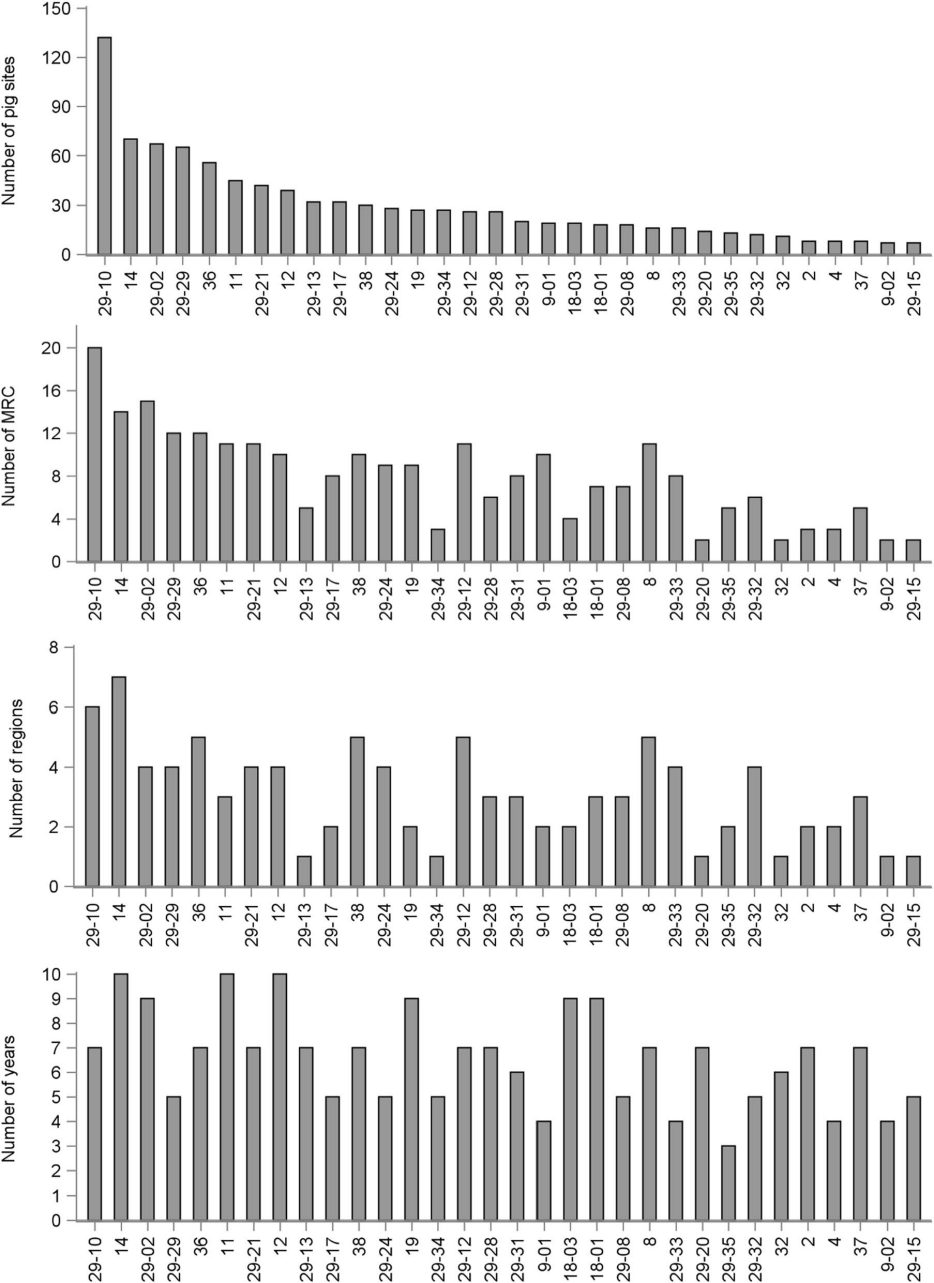
Number of pig sites, MRC, regions and years for each of the 32 wild-type clusters. A total of 1653 PRRSV ORF5 sequences from 693 pig sites located in 36 MRC (regional county municipalities) and 9 administrative regions in Quebec, Canada, were classified into 32 wild-type clusters for the study period (2010–2019)

The 32 wild-type clusters gathered a total of 1653 sequences from 693 distinct pig sites (Fig. 2) located in 36 MRC from 9 administrative regions. Each cluster was detected in a median number of 23 pig sites, ranging from 7 to 132 (Fig. 2). Five clusters (#29-10, 14, 29-02, 29-29, 36) were each detected on more than 50 pig sites. Each cluster was detected in up to 20 MRC and 7 administrative regions, although most of them were detected in ≤ 12 MRC (29/32; 91% of clusters) and ≤ 4 administrative regions (26/32; 81% of clusters) (Fig. 3). Finally, the median number of years for which clusters were detected over the follow-up period was 7, with seven clusters detected on ≥ 9 years (#14, 29-02, 11, 12, 19, 18-03, 18-01) (Fig. 3).

### Temporal distribution of wild-type genetic clusters

**Fig. 4:**
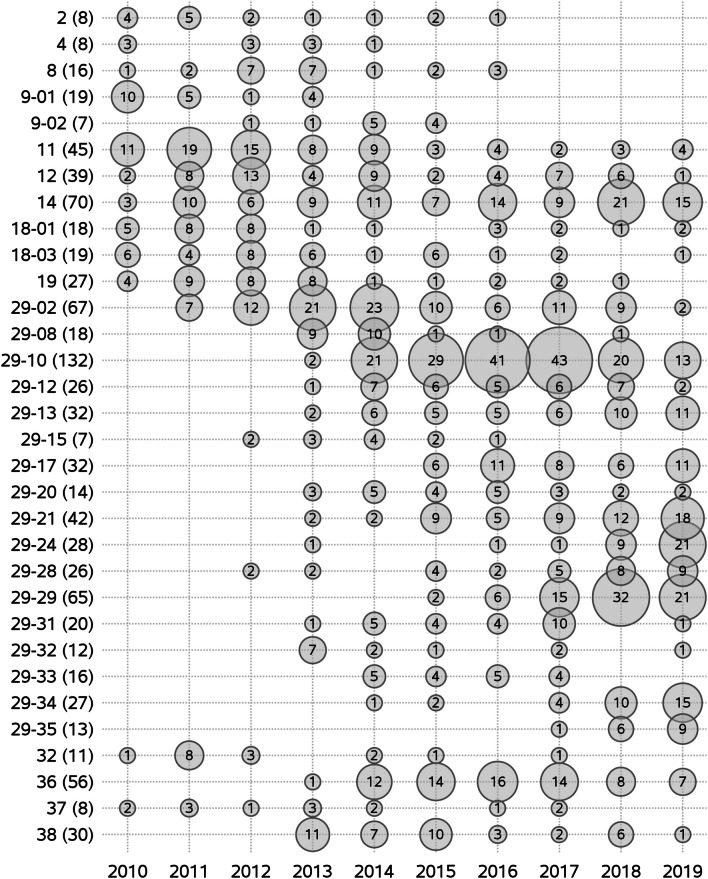
Number of pig sites for each of the 32 wild-type clusters according to year. A total of 1653 PRRSV ORF5 sequences from 693 pig sites in Quebec, Canada, were classified into 32 wild-type clusters for the study period (2010–2019). The number of sites in which the cluster was detected is shown between parentheses

The yearly number of pig sites in which each of the 32 wild-type clusters were detected is shown in Fig. 4. The maximum number of pig sites on which a cluster was detected within a year was 43. The mean (min–max) number of clusters detected in at least one pig site per year was 24 (17–29). The number of pig sites infected with new clusters derived from cluster #29 increased over the years (Fig. 4).

### Spatial distribution of wild-type genetic clusters

**Fig. 5:**
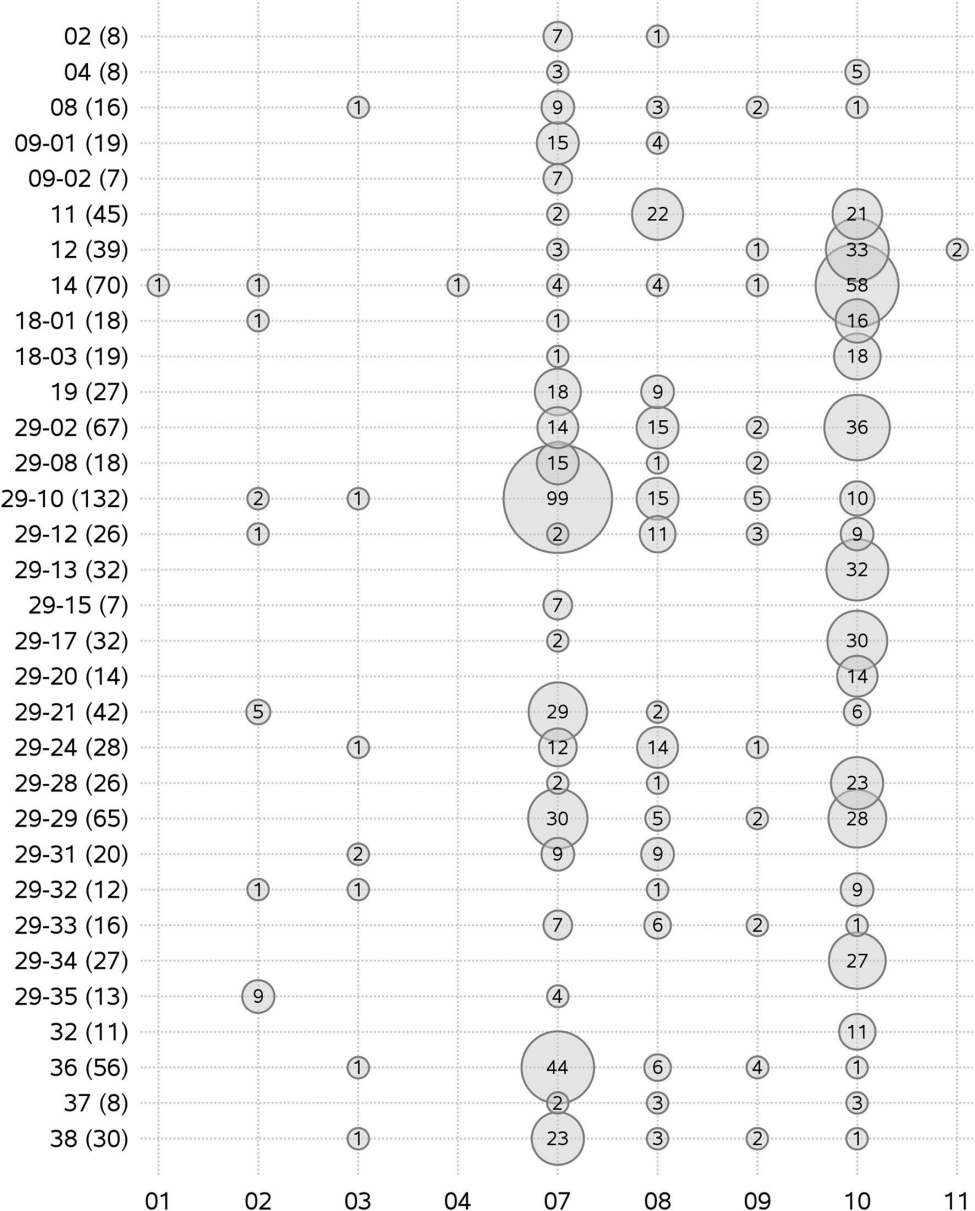
Number of pig sites for each of the 32 wild-type clusters according to administrative region. A total of 1653 PRRSV ORF5 sequences from 693 pig sites in Quebec, Canada, were classified into 32 wild-type clusters for the study period (2010–2019). The number of sites in which the cluster was detected during the study period is shown between parentheses. Administrative region codes are 01: Laurentides, 02: Lanaudiere, 03: Mauricie, 04: Capitale-Nationale, 07: Monteregie, 08: Centre-du-Quebec, 09: Estrie, 10: Chaudiere-Appalaches, 11: Bas St-Laurent

The number of pig sites in which each of the 32 wild-type clusters were detected per administrative region is displayed in Fig. 5. Whereas a large number of these clusters were observed in Monteregie (n = 27) and Chaudiere-Appalaches (n = 23) regions, other regions such as Bas St-Laurent, Laurentides and Capitale-Nationale had only one cluster detected (Fig. 5). Some clusters were predominantly detected in Chaudiere-Appalaches (#14, 29-13, 29-17, 29-34), whereas others were more predominant in Monteregie (#29-10, 29-21, 36), and Centre-du-Quebec shared many clusters with these two latter regions. Globally, administrative regions located in the north shore of the St. Lawrence River (e.g. Laurentides, Lanaudiere, Mauricie, and Capitale-Nationale) had a lower number of clusters (n = 12) detected compared to the others located in the south shore (n = 32).

### Spatiotemporal distribution of wild-type genetic clusters

**Fig. 6:**
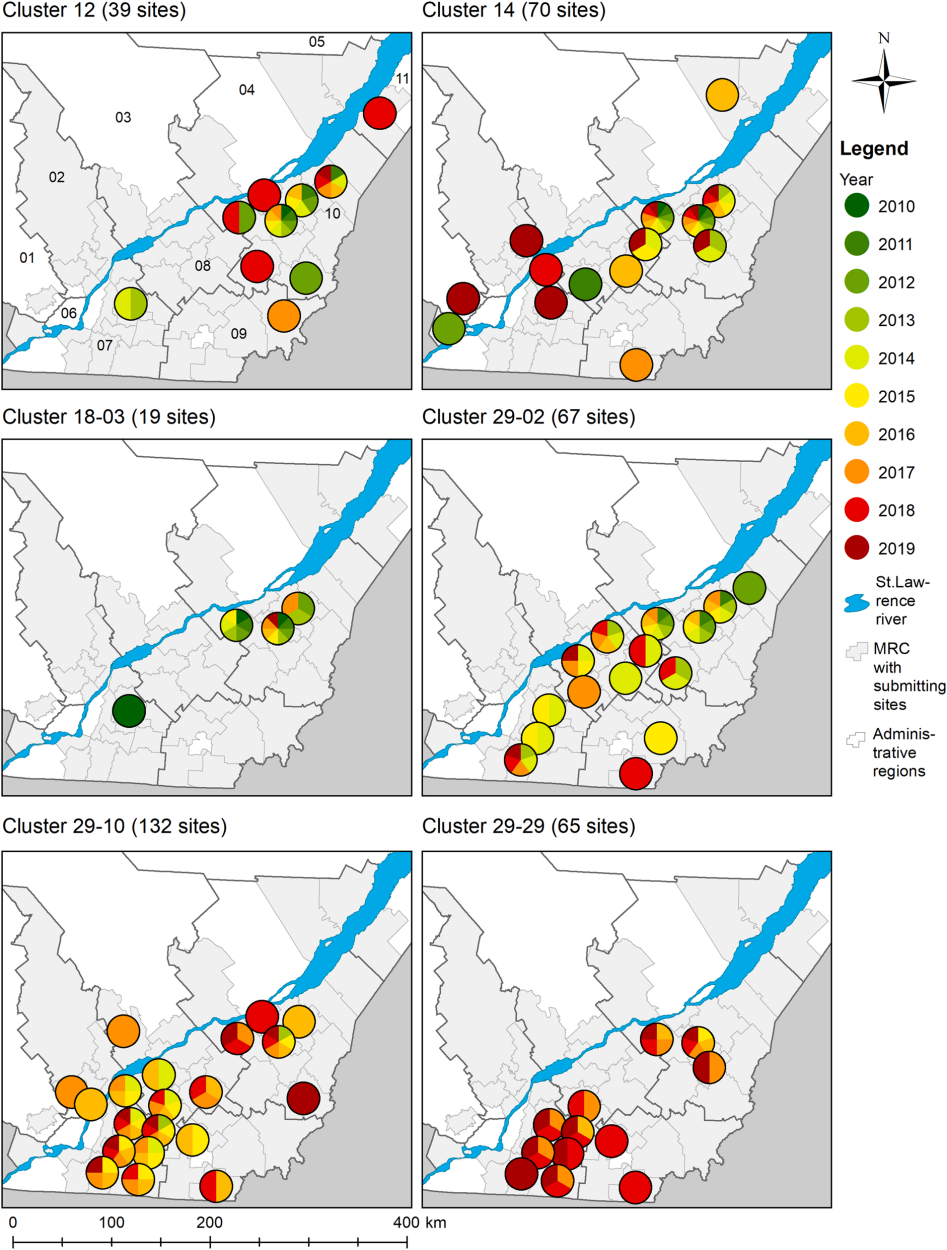
Maps of the spatiotemporal distributions of specific wild-type clusters. Each color in the pie chart indicates the detection of the cluster in at least one pig site located in the MRC during a specific year (2010–2019). The total number of sites in which a cluster was detected during the study period is shown between parentheses. Administrative region codes are 01: Laurentides, 02: Lanaudiere 03: Mauricie, 04: Capitale-Nationale 05: Saguenay-Lac-St-Jean, 06: Montreal-Laval, 07: Monteregie, 08: Centre-du-Quebec, 09: Estrie, 10: Chaudiere-Appalaches, 11: Bas St-Laurent

Different spatiotemporal distribution patterns were observed among the 32 wild-type clusters (Fig. 6 and Additional files 1, 2, 3, 4, 5, 6: Fig. S1-S6). Figure 6 shows a subset of the maps of the spatiotemporal distributions of six wild-type clusters, namely clusters #12, 14, 18-03, 29-02, 29-10 and 20-29. A first pattern corresponds to clusters observed in pig sites from a few MRC located in a maximum of 2 administrative regions over several years (e.g., Fig. 6 cluster #18-03 and Additional files 2 and 5, clusters #19 and #29-34, respectively). A second pattern is similar to the first one, except that detections were also occasionally noticed in a few MRC located away from the persistence centre (e.g., Fig. 6 cluster #12). A third pattern consists of clusters persisting throughout the years which are detected in ≥ 60 pig sites located in a large number of MRC from ≥ 4 administrative regions of the St. Lawrence River’s south shore (e.g., Fig. 6 clusters #29-02 and #29-29). Finally, a fourth pattern is similar to the third one except that some clusters were also found on the north shore (e.g., Fig. 6 clusters #14 and #29-10). Of the 12 clusters detected on pig sites located on the north shore, 10 (83%) were first detected on pig sites from the south shore and were later detected on the north shore.

Clusters #12, 14, 18-03, 29-02, 29-10 and 20-29.

### Genetic diversity analyses

**Fig. 7:**
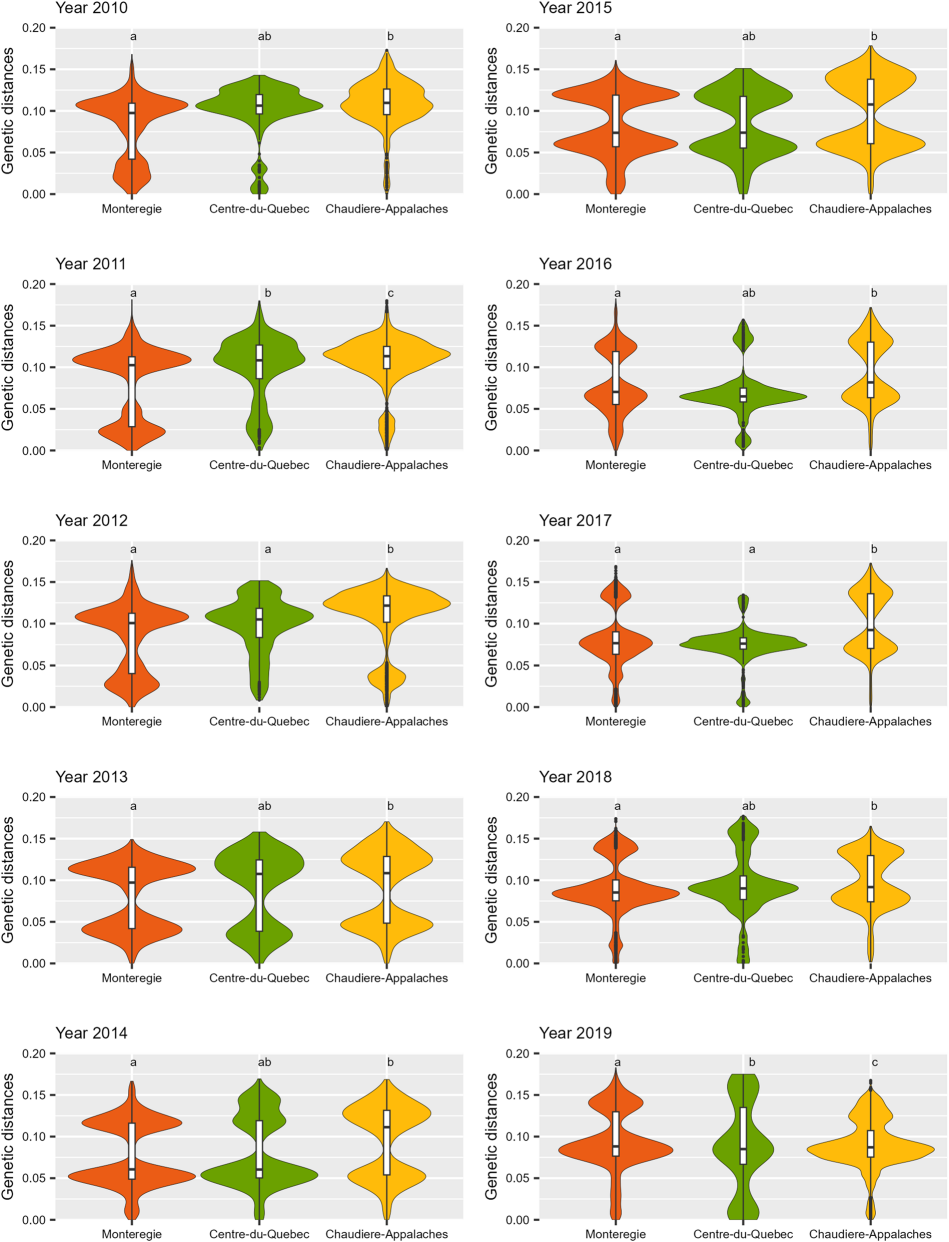
Distribution of genetic distances among wild-type sequences in three administrative regions according to year. A total of 2222 wild-type sequences (1 randomly selected per pig site and per year,) submitted between 2010 and 2019 were included. Within a specific year, regions with different letters indicate a significant pairwise difference in genetic diversity from the molecular analysis of variance (AMOVA) (*P* < 0.002), considering a Bonferroni adjustment for multiple testing. Monteregie, orange; Centre-du-Quebec, green; Chaudiere-Appalache, yellow

Three administrative regions, Monteregie, Centre-du-Quebec and Chaudiere-Appalaches, had more than 5 pig sites with wild-type sequences for each year of the study period. Overall, 2434 wild-types sequences from 946 pig sites were obtained in these three regions. From them, 2222 sequences were randomly selected (1 per site per year) for genetic diversity analyses. The distribution of genetic distances according to each of these three administrative regions per year is shown in Fig. 7. A significant difference between regions in genetic diversity of wild-type sequences was observed in each year (*P* < 0.005) (Fig. 7). Post-hoc comparisons revealed that genetic diversity in Chaudiere-Appalaches was higher than in Monteregie (*P* ≤ 0.002) for each year of the study and than in Centre-du-Quebec for four years (2011, 2012, 2017, 2019). The genetic diversity in the Centre-du-Quebec compared to that in Monteregie was higher in 2011 but lower in 2019. Other pairwise comparisons were not significant.

## Discussion

Within herd PRRSV sequencing was requested by the herd veterinarian for a variety of reasons including the diagnostic of PRRS, the assessment of genetic evolution in endemic strains or the monitoring of an elimination process. Even if monetary incentives from the Éleveurs de porcs du Québec and the Quebec Department of Agriculture, Fisheries and Food (MAPAQ) were provided to promote sequencing for some of the years studied, sequencing was always performed on a producer voluntary basis. Consequently, the yearly average of 329 pig sites most likely represents only a part of PRRSV positive pig sites. More specifically, herds harboring persistent strains but without exhibiting significant clinical signs might have been less likely to submit samples for PRRSV sequencing. In addition, since the long-term impact of the disease and thus the incentive for diagnostic is greater in breeding sites compared to growing or finishing sites, some of the latter could be missing in our study population. Nevertheless, since several PRRS ARC&E initiatives were ongoing during the study period throughout the province, most PRRSV clinical outbreaks would probably have led to requests for sequencing. In addition, the provincial PRRSV sequence sharing agreement signed in 2010 by all swine practitioners allows the transfer of all sequences obtained from the three diagnostic laboratories to the LEMP-DB for surveillance and research purposes.

Unlike other studies which focused on a single or on few clusters [13] or subgroups or lineages [14, 22, 34] or limited to a few farms [8] our study covered all wild-type clusters found over a 10 year-period at the provincial level. Furthermore, each cluster was described based on its detection in at least one of pig site within a year instead of the number of detections, in order to prevent the influence of repeated sampling of the same strain within a herd during the same year. The detection of a PRRSV strain in a pig site could result from the sampling of sites chronically infected with an endemic strain or from a new introduction. All these infected sites represent a potential source of infection for other pig sites for at least one part of the year, even though the risk may vary according to the level of contacts with other sites and the duration of virus shedding [32].

The 32 wild-type clusters obtained from 1653 sequences illustrates the considerable diversity at the provincial scale. Since vaccine-like sequences originate from the wide use of PRRS commercial vaccines, they were not included here. Moreover, since many sequences were not included in these clusters (Fig. 2), the number of clusters underestimated the total amount of diversity found at the provincial level. These results are in agreement with previous studies on PRRSV diversity. Most clusters detected between 1998 and 2016 [19] were still detected during the current study period (2010–2019), showing their persistence over at least two decades at the provincial level. Some clusters (#9, 18, 29), as they became larger and more diversified over time, were divided into several clusters. The detection of new cluster(s) at the provincial level could be due to the genetic evolution of PRRSV populations through natural selection, fitness, dynamics of adaptation, etc. From a genetic point of view, it would be interesting to evaluate if those clusters had higher mutation rate(s) compared to others, and what mechanisms might be driving the differences in these mutation rates. Conversely, the absence of detection of a specific cluster could be indicative of their successful elimination in the field or the absence of sampling among infected herds.

On the 12 administrative regions housing swine production in Quebec, ten had pig sites with available PRRSV ORF5 sequencing data (Fig. 1). According to the Veille Sanitaire Provinciale database, the two remaining regions, Saguenay-Lac-Saint-Jean and Abitibi-Temiscamingue, were home to only 11 and 9 pig sites, respectively in 2021. Most swine production in Quebec is located in the south shore of the St. Lawrence River, and more specifically within three administrative regions (i.e., Monteregie, Chaudiere-Appalaches and Centre-du-Quebec), and thus provided the bulk on sequences analyzed and the larger number of clusters detected. This highlights the potential risk of transmission of PRRSV strains among pig sites within these regions and thus challenges in controlling the disease. Even if the number of clusters detected was higher in Monteregie (n = 27) than Chaudiere-Appalaches (n = 23) (Fig. 5), the molecular analysis of variance indicates a higher level of diversity in Chaudiere-Appalaches compared to Monteregie, for each year of the study (Fig. 7). This shows the importance to avoid using solely the number of clusters to interpret the level of diversity within a region since it can be influenced by the number of sites of origin, the number of unclassified sequences, and the genetic distances between clusters. The proportion of pig sites part of a vertically integrated system is larger in Monteregie than in Chaudiere-Appalaches which is housing more independently owned pig sites. Vertically integrate system, using multi-site productions, might favor the transmission of a particular cluster to several pig sites through pig movements. Consequently, the detection of clusters having 15 sequences or more could be higher compared to independent pig sites. Further studies are nevertheless required to test this hypothesis.

Even if some clusters were observed in both Monteregie and Chaudiere-Appalaches, these two regions exhibited important differences in terms of predominant clusters (Fig. 5). Then, keeping those regions into separated control zones would be advisable in order to facilitate PRRSV regional control. By opposition, the Centre-du-Quebec often shared clusters with both adjacent regions, Monteregie and Chaudiere-Appalaches, suggesting the presence of different network connections between pig sites (e.g., pig movement, personnel, visitors, service vehicles) of both regions [20], which could complicate disease control.

Spatiotemporal analyses revealed large variations in the distribution of wild-type clusters at the provincial scale. Whereas some clusters remained localized, others were detected on a larger number of sites and MRCs over time. The current results are nonetheless helpful to guide different stakeholders in planning control initiatives at the provincial scale. In fact, considering that 94% of the 32 clusters were detected first on the south shore and that regions from the north shore (e.g., Laurentides, Lanaudiere, Mauricie, and Capitale-Nationale) remained free from 60% of 32 clusters over a 10-year period, the north shore might be more suitable to a regional elimination attempt, by limiting pig movements from the South to the north shore. Cautious is nevertheless advisable since many sequences were not included in the 32 clusters kept for analyses.

Since the sequencing methodology used in our study can only detect a single PRRSV sequence per sample, only the predominant sequence was likely identified. This methodology may have prevented the detection of some wild-type strains in herds where PRRSV-vaccine like sequences were found. As the ORF5 gene covers only 4% of the whole PRRSV genome, it is possible that whole genome sequencing might have generated different clustering results. In addition, whole genome sequencing might have revealed recombination amongst wild-type strains, and between wild-type and vaccine-like strains. They might have revealed even more complexity in the spatiotemporal distribution of PRRSV sequences. The MRC spatial units were chosen for a first exploration of the patterns and to produce outputs easily understandable and meaningful for the different industry stakeholders. Visual patterns could have been different if other geographical units had been used for representation. In addition, since the mapping is based on the presence of at least one infected pig site instead of the actual number of infected sites, the regional risk may vary between positive MRCs. Here, because clusters were not associated to clinical data, the levels of distribution of some clusters do not necessarily reflect their economic impacts.

As next steps, phylogeographic analysis would allow an evaluation of dispersion patterns including direction and speed of the transmission between sites. Many reasons might explain the differences observed in the distributions of clusters including virus characteristics or the different network connections between pig sites [20]. Clearly, attempts to successfully limit the number of sites infected should include a thorough assessment of the factors responsible for the rapid dissemination of specific clusters over time.

## Conclusions

The considerable PRRSV diversity observed in the province of Quebec during our study period is certainly a major reason for the very challenging management of the disease. The ongoing molecular-based surveillance system highlighted differences in both the spatiotemporal distributions of PRRSV clusters and in the regional diversity of wild-type sequences. Reasons behind these differences need to be further explored in order to get insight to improve collective PRRS management strategies.

### Supplementary Information

**Additional file 1: Fig. S1. **Spatiotemporal distribution of clusters #2, 4, 8, 9-01 and 9-02.

**Additional file 2: Fig. S2. **Spatiotemporal distribution of clusters #11, 12, 14, 18-01, 18-03 and 19.

**Additional file 3: Fig. S3. **Spatiotemporal distribution of clusters #29-02, 29-08, 29-10, 29-12, 29-13 and 29-15.

**Additional file 4: Fig. S4. **Spatiotemporal distribution of clusters #29-17, 29-20, 29-21, 29-24, 29-28 and 29-29.

**Additional file 5: Fig. S5. **Spatiotemporal distribution of clusters #29-31, 29-32, 29-33, 29-34, 29-35 and 32.

**Additional file 6: Fig. S6. **Spatiotemporal distribution of clusters #36, 37 and 38.

**Additional file 7: **Legends for Additional files 1 to 6: Figs. S1 to S6. Spatiotemporal distribution of specific wild-type clusters.

## Data Availability

The datasets analysed in our study are not publicly available. They are covered by confidentiality agreements between the swine production sites, the veterinarians, the diagnostic laboratories and the LEMP. They were required for the transfer of sequences and data from the diagnostic laboratories to the LEMP-DB.

## Abbreviations

ARC&E: Area-based regional control and elimination
LEMP: Laboratoire d’épidémiologie et de médecine porcine
LEMP-DB: Laboratoire d’épidémiologie et de médecine porcine - database
ORF5: Open reading frame 5
PRRS: Porcine reproductive and respiratory syndrome
PRRSV: Porcine reproductive and respiratory syndrome virus
