# Supplementary figures and images for: A descriptive study on spatial and temporal distributions of genetic clusters of porcine reproductive and respiratory syndrome virus infecting pig sites in Quebec, Canada, between 2010 and 2019
Source: Porcine Health Manag. 2024 Jan 25;10:7. doi: 10.1186/s40813-024-00357-x (PMC10809575; doi:10.1186/s40813-024-00357-x)

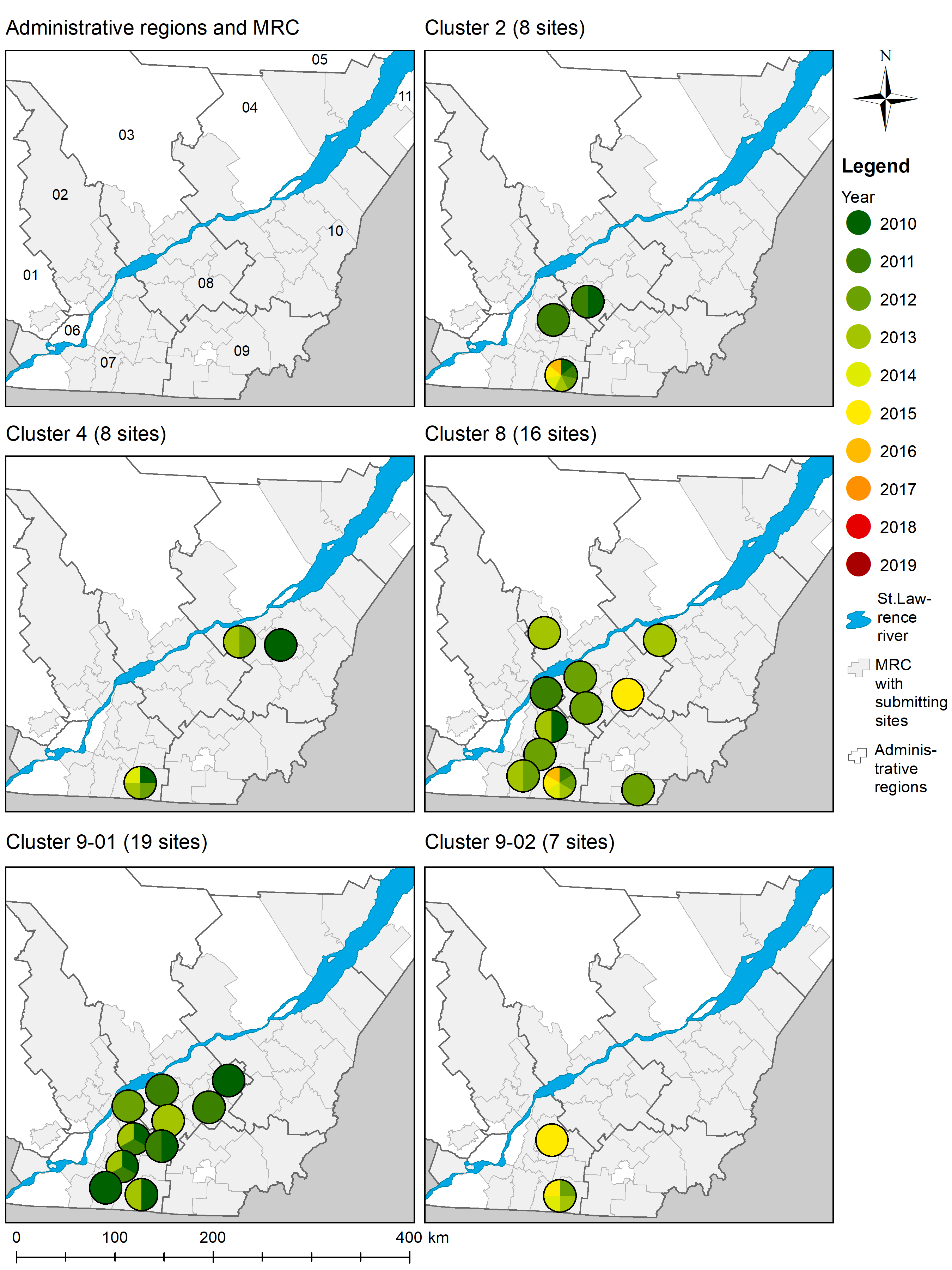

Supplement: Supplementary file 1 — Additional file 1: Fig. S1. Spatiotemporal distribution of clusters #2, 4, 8, 9-01 and 9-02. [file 40813_2024_357_MOESM1_ESM.tif]

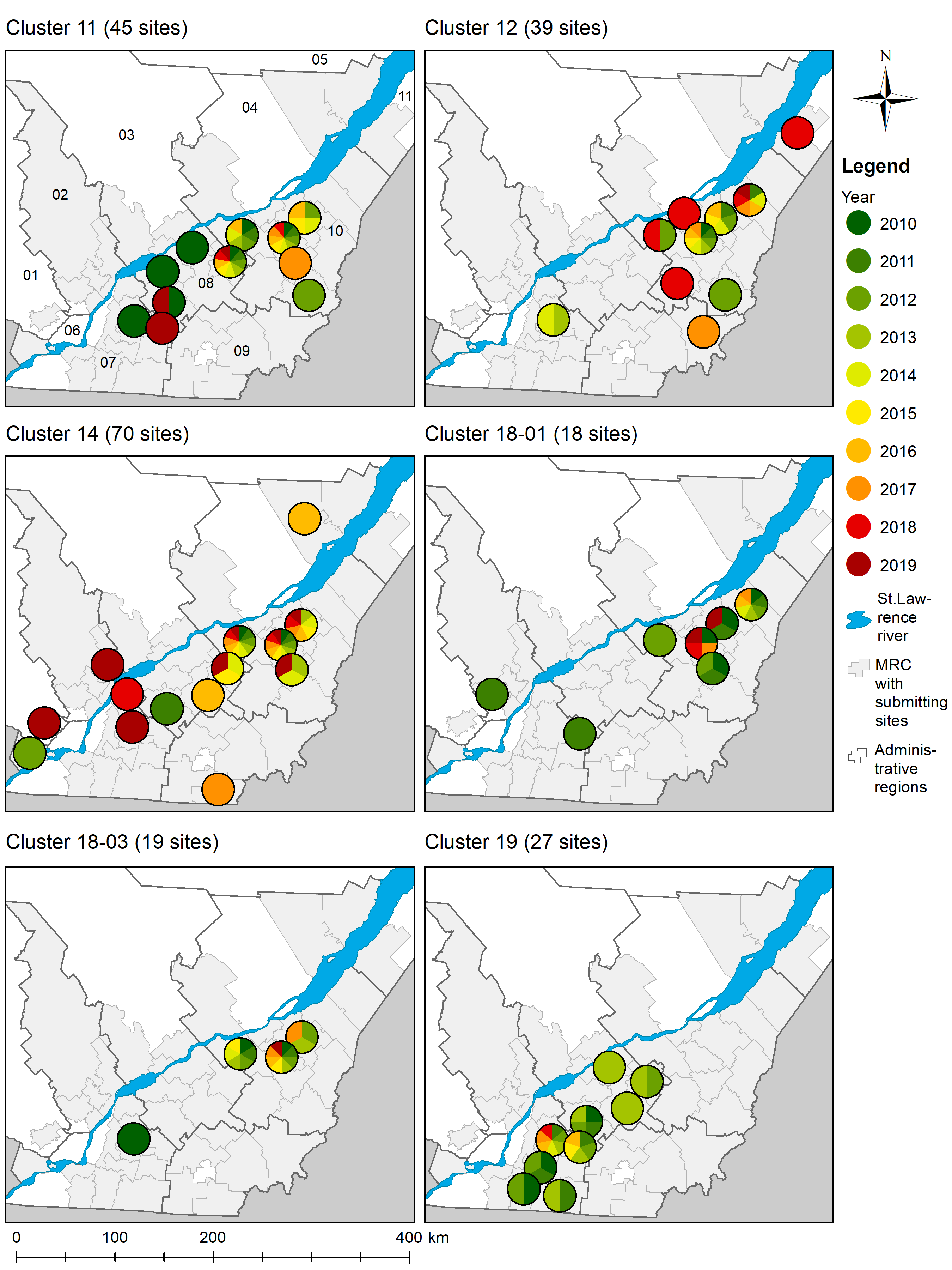

Supplement: Supplementary file 2 — Additional file 2: Fig. S2. Spatiotemporal distribution of clusters #11, 12, 14, 18-01, 18-03 and 19. [file 40813_2024_357_MOESM2_ESM.tif]

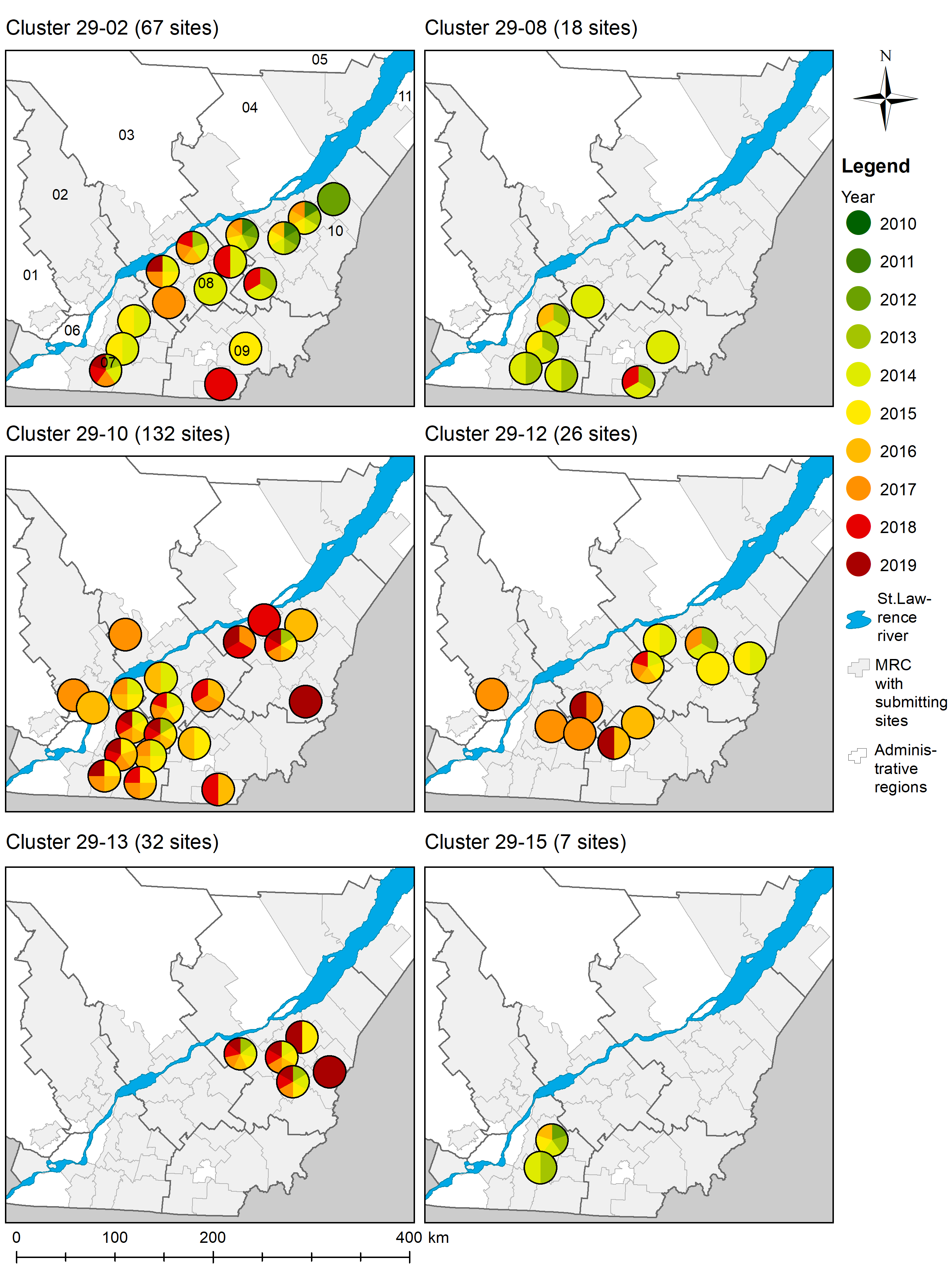

Supplement: Supplementary file 3 — Additional file 3: Fig. S3. Spatiotemporal distribution of clusters #29-02, 29-08, 29-10, 29-12, 29-13 and 29-15. [file 40813_2024_357_MOESM3_ESM.tif]

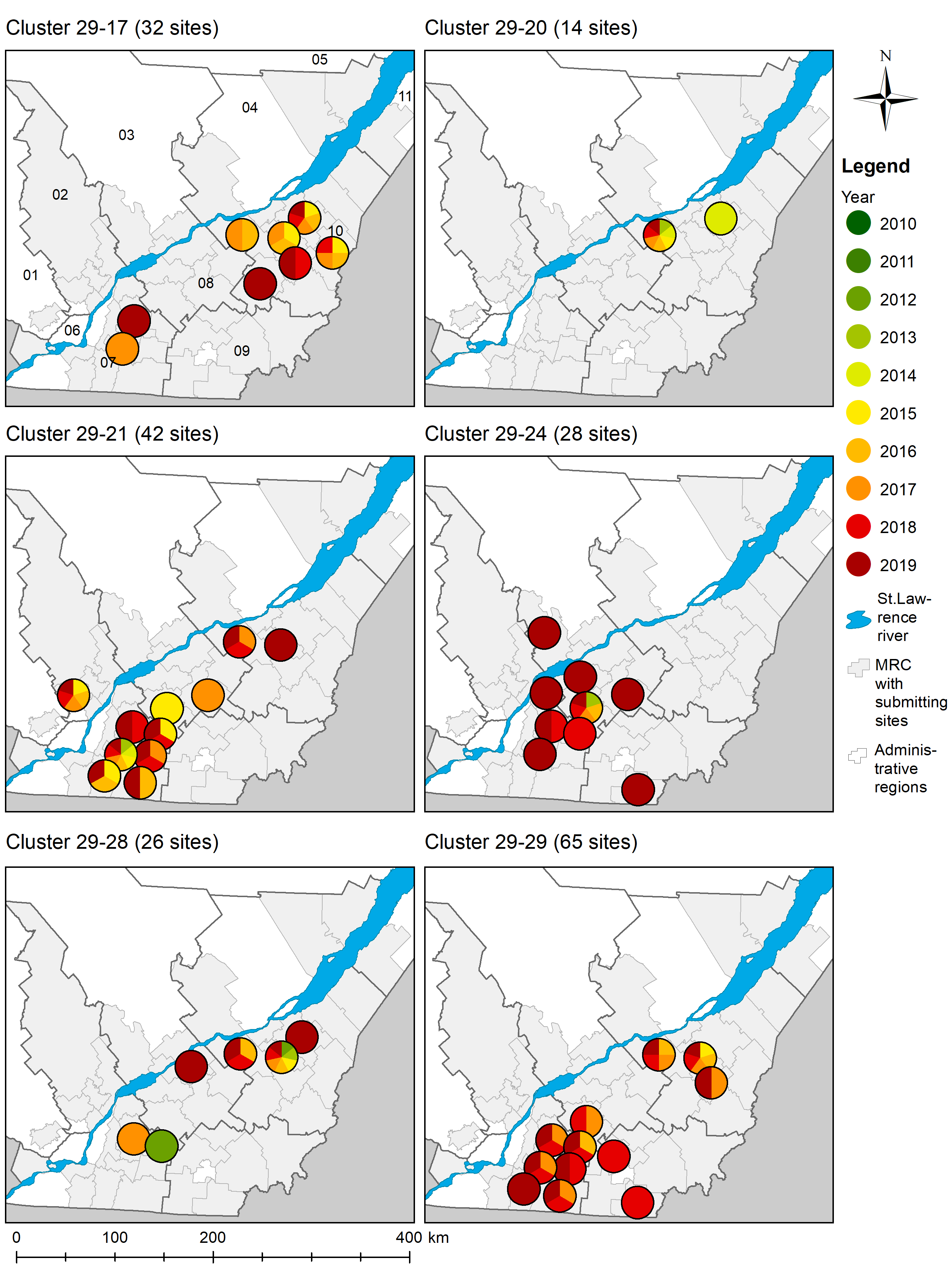

Supplement: Supplementary file 4 — Additional file 4: Fig. S4. Spatiotemporal distribution of clusters #29-17, 29-20, 29-21, 29-24, 29-28 and 29-29. [file 40813_2024_357_MOESM4_ESM.tif]

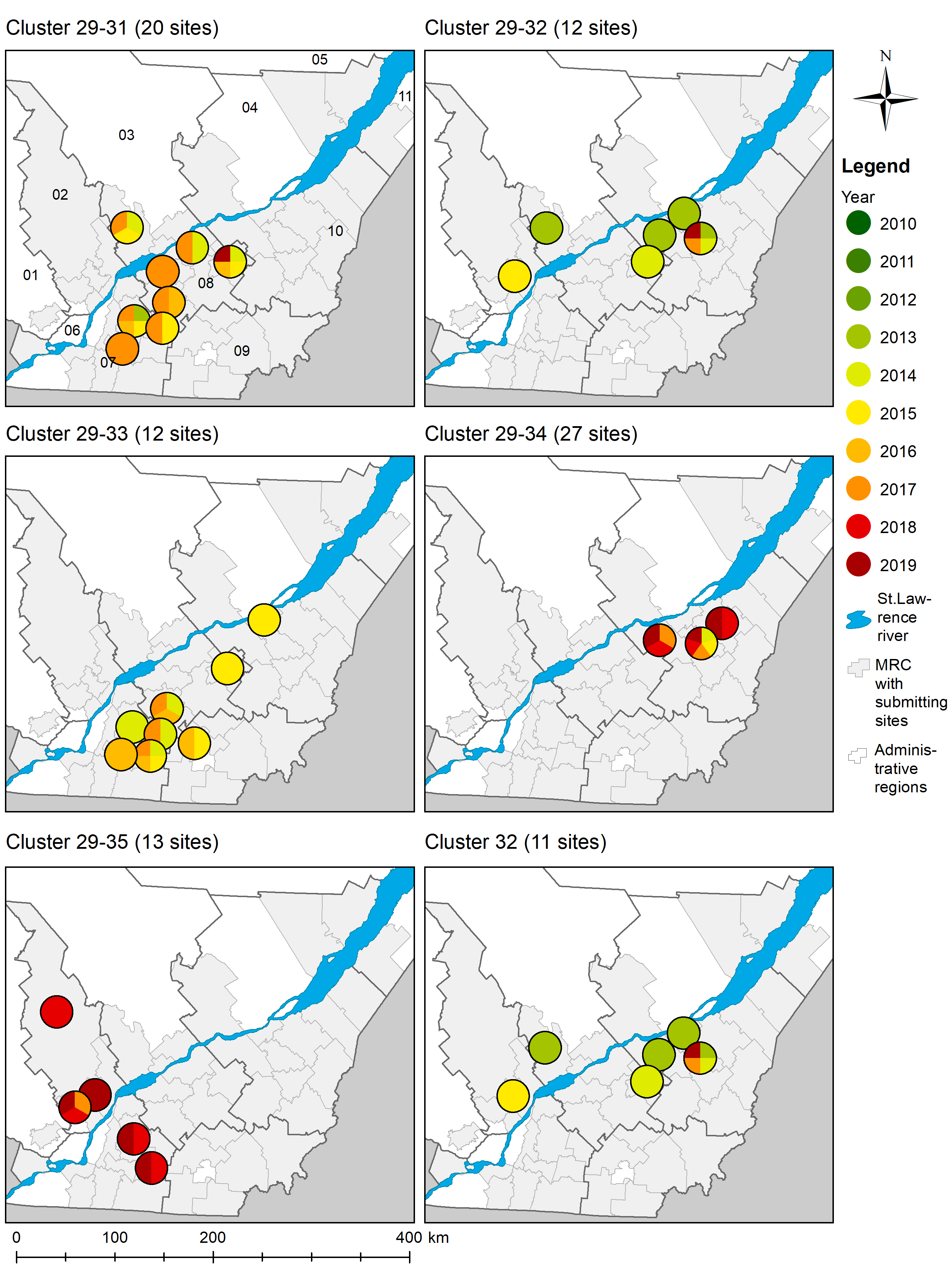

Supplement: Supplementary file 5 — Additional file 5: Fig. S5. Spatiotemporal distribution of clusters #29-31, 29-32, 29-33, 29-34, 29-35 and 32. [file 40813_2024_357_MOESM5_ESM.tif]

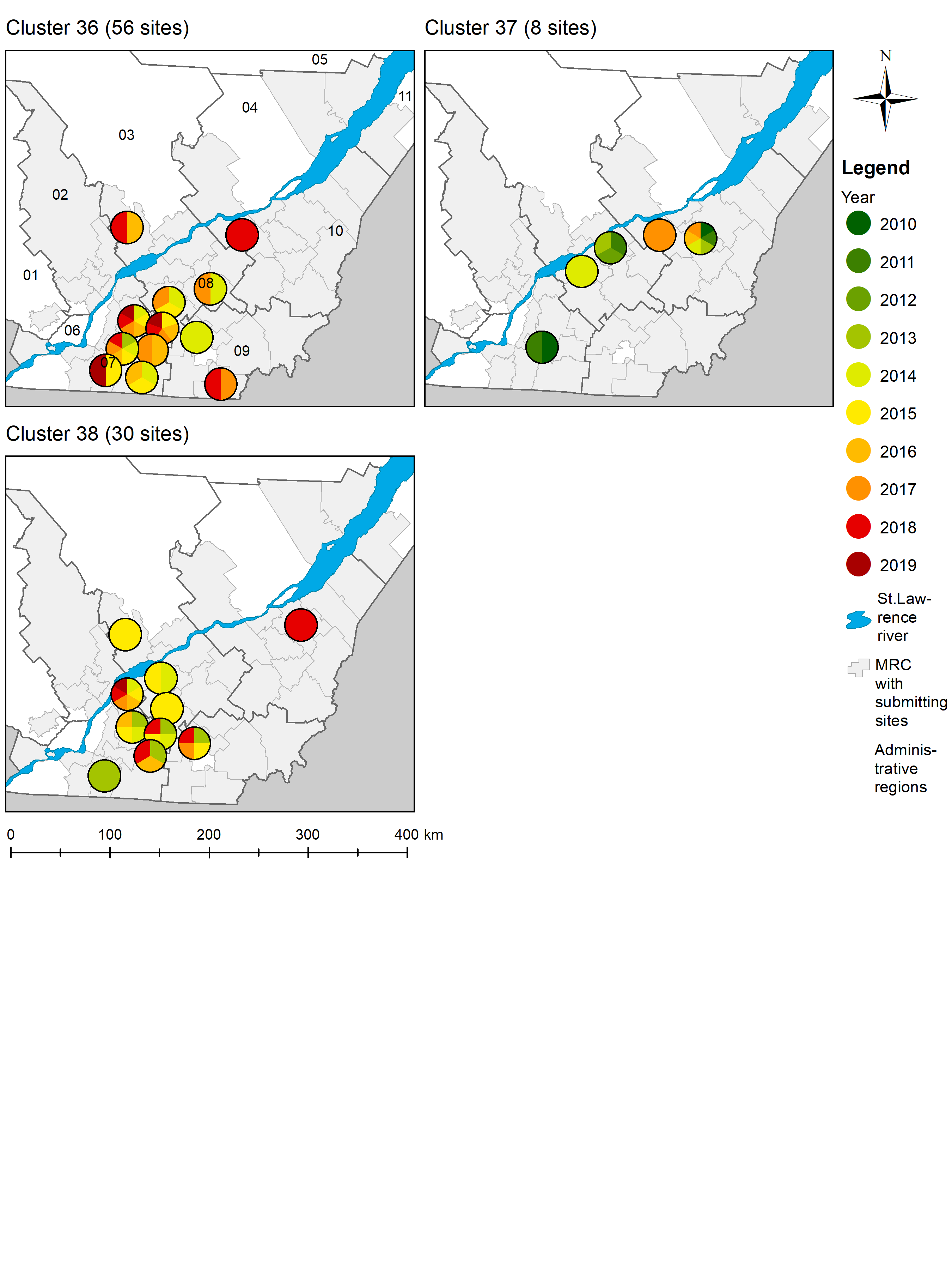

Supplement: Supplementary file 6 — Additional file 6: Fig. S6. Spatiotemporal distribution of clusters #36, 37 and 38. [file 40813_2024_357_MOESM6_ESM.tif]
