# Supplementary material for: A descriptive study on spatial and temporal distributions of genetic clusters of porcine reproductive and respiratory syndrome virus infecting pig sites in Quebec, Canada, between 2010 and 2019
Source: Porcine Health Manag. 2024 Jan 25;10:7. doi: 10.1186/s40813-024-00357-x (PMC10809575; doi:10.1186/s40813-024-00357-x)
Supplement: Supplementary file 7 — Additional file 7: Legends for Additional files 1 to 6: Figs. S1 to S6. Spatiotemporal distribution of specific wild-type clusters. [file 40813_2024_357_MOESM7_ESM.docx]

Additional file 7: Legends for Additional files 1 to 6: Figs. S1 to S6. Spatiotemporal distribution of specific wild-type clusters.

Each color in the pie chart indicates the detection of the cluster in at least one pig site located in the MRC during a specific year (2010-2019). The total number of sites in which a cluster was detected during the study period is shown between parentheses. Administrative region codes are 01: Laurentides, 02: Lanaudiere 03: Mauricie, 04: Capitale-Nationale 05: Saguenay-Lac-St-Jean, 06: Montreal-Laval, 07: Monteregie, 08: Centre-du-Quebec, 09: Estrie, 10: Chaudiere-Appalaches, 11: Bas St-Laurent.

Additional file 1: Fig. S1. Clusters #2, 4, 8, 9-01, 9-02.

Additional file 2: Fig. S2. Clusters #11, 12, 14, 18-01, 18-03, 19.

Additional file 3: Fig. S3. Clusters #29-02, 29-08, 29-10, 29-12, 29-13, 29-15.

Additional file 4: Fig. S4. Clusters #29-17, 29-20, 29-21, 29-24, 29-28, 29-29.

Additional file 5: Fig. S5. Clusters #29-31, 29-32, 29-33, 29-34, 29-35, 32.

Additional file 6: Fig. S6. Clusters #36, 37, 38.
